# Follistatin as a Potential Biomarker for Identifying Metabolically Healthy and Unhealthy Obesity: A Cross-Sectional Study

**DOI:** 10.3390/jpm14050487

**Published:** 2024-05-03

**Authors:** Ayşe N. Erbakan, H. Hicran Mutlu, Mehmet Uzunlulu, Lütfullah Caştur, Muhammet Mikdat Akbaş, Fatoş N. Kaya, Mehmet Erbakan, Ferruh K. İşman, Aytekin Oğuz

**Affiliations:** 1 Department of Internal Medicine, Istanbul Medeniyet University, Goztepe Prof. Dr. Suleyman Yalcin City Hospital, Kadikoy, 34722 Istanbul, Turkey; 17330803003@ismu.edu.tr (A.N.E.); mehmet.uzunlulu@medeniyet.edu.tr (M.U.); muhammetmikdat@gmail.com (M.M.A.); nimet.dolu@gmail.com (F.N.K.); aytekinoguz@hotmail.com (A.O.); 2 Department of Family Medicine, Istanbul Medeniyet University, Goztepe Prof. Dr. Suleyman Yalcin City Hospital, Kadikoy, 34722 Istanbul, Turkey; hicran.mutlu@medeniyet.edu.tr; 3 Department of Internal Medicine, Istanbul Mehmet Akif Ersoy Thoracic and Cardiovascular Surgery Training and Research Hospital, 34303 Istanbul, Turkey; lcastur@hotmail.com; 4 Department of Family Medicine, Health Sciences University, Kartal Dr. Lutfi Kirdar City Hospital, Kartal, 34865 Istanbul, Turkey; 5 Department of Biochemistry, Istanbul Medeniyet University, Goztepe Prof. Dr. Suleyman Yalcin City Hospital, Kadikoy, 34722 Istanbul, Turkey; ferruh.isman@medeniyet.edu.tr

**Keywords:** follistatin, metabolically healthy obesity, metabolically unhealthy obesity, metabolic syndrome, obesity, metabolic health, biomarker

## Abstract

Metabolically healthy obesity (MHO) refers to obese individuals with a favorable metabolic profile, without severe metabolic abnormalities. This study aimed to investigate the potential of follistatin, a regulator of metabolic balance, as a biomarker to distinguish between metabolically healthy and unhealthy obesity. This cross-sectional study included 30 metabolically healthy and 32 metabolically unhealthy individuals with obesity. Blood samples were collected to measure the follistatin levels using an enzyme-linked immunosorbent assay (ELISA). While follistatin did not significantly differentiate between metabolically healthy (median 41.84 [IQR, 37.68 to 80.09]) and unhealthy (median 42.44 [IQR, 39.54 to 82.55]) individuals with obesity (*p* = 0.642), other biochemical markers, such as HDL cholesterol, triglycerides, C-peptide, and AST, showed significant differences between the two groups. Insulin was the most significant predictor of follistatin levels, with a coefficient of 0.903, followed by C-peptide, which exerted a negative influence at −0.624. Quantile regression analysis revealed nuanced associations between the follistatin levels and metabolic parameters in different quantiles. Although follistatin may not serve as a biomarker for identifying MHO and metabolically unhealthy obesity, understanding the underlying mechanisms that contribute to metabolic dysfunction could provide personalized strategies for managing obesity and preventing associated complications.

## 1. Introduction

Obesity is an important contributor to the incidence and severity of chronic diseases such as type 2 diabetes mellitus (T2DM) and cardiovascular disease (CVD) [1]. Addressing obesity is, therefore, an important and urgent global public health issue. Metabolically healthy obesity (MHO) is a term used to define a specific group of people who are obese and have an acceptable metabolic profile characterized by the absence of metabolic syndrome [2]. MHO has recently gained increasing importance in the field of obesity-related diseases as it may be relevant to certain public health approaches and strategies.

The presence of MHO varies considerably among those classified as obese, ranging from 6.8% to 36.6% [3]. Several studies have characterized MHO as individuals who are obese but have increased insulin sensitivity, without concomitant diabetes, dyslipidemia, or hypertension [4]. Although MHO is recognized as an individual phenotype, there is an ongoing debate about its therapeutic relevance and long-term complications. The conflicting data in the scientific literature raise skepticism about the claim that MHO does not pose a health risk, as there is a concern that it can progress to metabolically unhealthy obesity (MUO) in the long term. The results of four studies involving 23,209 people and a follow-up period of at least 10 years revealed that metabolically healthy but obese individuals have a 24% increased likelihood of experiencing all-cause mortality or cardiovascular events compared to normal-weight people with no metabolic risk factors [5]. Some data also suggest that MHO is not necessarily a harmless condition in terms of cardiovascular and metabolic health [6,7]. In another study, metabolically healthy obese individuals had a 30–50% lower risk of all-cause mortality, non-fatal and fatal cardiovascular disease, and cancer mortality than metabolically unhealthy obese individuals after adjusting for fitness and other confounders [8].

Adipokines, which are biologically active chemicals released by adipose tissue, have gained attention as regulators of metabolic balance. Among these, follistatin, previously known for its reproductive function, has attracted interest owing to its involvement in adipogenesis and metabolic control [9]. Follistatin expression has been observed to change significantly in various metabolic disorders, such as obesity and type 2 diabetes mellitus (T2DM), suggesting a possible link between its dysregulation and metabolic health [10]. Research has shown that follistatin acts as a pathological hepatokine that can be targeted for diabetes treatment in hepatic insulin resistance [11]. Furthermore, follistatin has been found to inhibit myostatin, a protein that negatively regulates muscle growth [12]. This suggests that follistatin may also play a role in muscle metabolism and the overall body composition. Studies have shown that elevated follistatin levels are associated with improved glucose metabolism and increased insulin sensitivity, suggesting a potential therapeutic target for metabolic disorders. Short-term administration of follistatin decreases glucagon secretion from Langerhans islets, whereas long-term administration prevents apoptosis and stimulates the proliferation of β-cells in rats [13]. Local overexpression of follistatin in the pancreas of diabetic mice leads to an increase in the serum insulin levels [14]. In humans, follistatin is primarily derived from the liver, and its expression and secretion are upregulated by a high ratio of glucagon to insulin [11]. Understanding the complex role of follistatin in metabolism may lead to the development of new therapies for obesity and T2DM.

This study aimed to investigate the emerging importance of follistatin as a potential biomarker for metabolically healthy and unhealthy obesity. Furthermore, this study investigated the correlation between follistatin and various biochemical assays, such as insulin levels, C-peptide levels, HDL cholesterol levels, and age.

## 2. Materials and Methods

This cross-sectional study included individuals with obesity, defined as a body mass index ≥ 30, who visited outpatient clinics between 15 January and 15 July 2022. The study protocol was approved by the Ethics Committee of the Istanbul Medeniyet University Goztepe Training and Research Hospital (No.2021/0673) and was carried out in accordance with the Declaration of Helsinki. All the participants provided informed consent. The clinical trial registration number is NCT06229899.

Metabolic syndrome was diagnosed when a patient had at least three of the following five conditions: fasting blood glucose (FBG) levels ≥ 100 mg/dL (or current drug therapy for hyperglycemia), blood pressure levels ≥ 130/85 mmHg (or current drug therapy for hypertension), triglyceride levels ≥ 150 mg/dL (or current drug therapy for hypertriglyceridemia), HDL cholesterol (HDL-C) levels below 40 mg/dL in men or below 50 mg/dL in women (or current drug therapy for reduced HDL-C), and a waist circumference of ≥102 cm in men or ≥88 cm in women. Individuals who did not meet any criteria for metabolic syndrome other than an increased waist circumference were defined as having metabolically healthy obesity, while those with ≥1 criterion for metabolic syndrome other than an increased waist circumference were defined as having metabolically unhealthy obesity [7]. To exclude prediabetes, the MHO group also had an HbA1c level <6%.

Inclusion and exclusion criteria: Individuals with obesity were eligible if they were between 18 and 60 years of age and had a BMI ≥ 30 kg/m^2^. Individuals taking antihypertensive medications, receiving lipid-lowering therapy, pregnant women, those with uncontrolled hypothyroidism or hyperthyroidism, steroid use, benign or malignant tumors, liver damage, and individuals who had secondary causes of obesity were not included.

Primary endpoint: The primary endpoint of this study was to assess the role of the follistatin level in distinguishing between metabolically healthy and unhealthy obesity.

Secondary endpoint: The secondary endpoint of the study was to identify biomarkers that influence follistatin levels. 

Sociodemographic information, comorbidities, previous and current medication use, and smoking and alcohol consumption habits were also recorded. The fasting blood glucose (FBG), HbA1c, and insulin levels were measured, along with the lipid panel and AST, ALT, urea, and creatinine levels. Furthermore, a complete blood count and the serum TSH levels, as well as the sT3, sT4, and cortisol levels, were determined. Anthropometric measurements, such as height, weight, and waist circumference, were obtained using standard instruments. The body mass index (BMI) was calculated as weight (kg) divided by height (m^2^).

### 2.1. Analytical Measurements

All the blood tests were performed after 10–14 h of fasting in the central hospital laboratory. The hexokinase technique was used to determine the fasting blood glucose (FBG) concentrations. The kinetic Jaffe technique was used to measure the serum creatinine levels. An enzymatic (without P-5′-P, NADH) technique was employed to determine the ALT concentrations. Enzymatic methods were used to quantify the fasting plasma total cholesterol, HDL and LDL cholesterol, and triglyceride concentrations (Abbott Architect c16000 and c8000; Abbott). For the HbA1c measurements, a Tosoh HLC-723 G8 (Tosoh G8) (variant-mode) ion-exchange high-performance liquid chromatography (HPLC) system (Tosoh, Tokyo, Japan) was used.

The blood samples were stored at −80 degrees Celsius and the follistatin levels were measured and analyzed using ELISA.

### 2.2. Statistical Analysis

In this study, the normality of the numerical variables was assessed using the Kolmogorov–Smirnov test. Normally distributed variables were presented as the mean ± standard deviation (SD), while non-normal distributed variables were presented as the median (interquartile range, IQR). Categorical variables were summarized using the frequency and percentage metrics.

To analyze the differences between the groups regarding the categorical variables, the Pearson chi-square test was used when the expected frequencies exceeded 5 in the 2 × 2 tables. Conversely, Fisher’s exact test was used when the expected frequency was less than 5. In comparisons involving two independent groups, Student’s *t*-test was employed for variables demonstrating a normal distribution, while the Mann–Whitney U test was applied in cases where a normal distribution was not observed. A multivariate linear regression analysis was performed to explore the individual and interaction effects of the independent variables on the dependent variable. Additionally, quantile regression was used to elucidate how the relationships between the variables varied across distinct quantiles of the dependent variable. Statistical significance was established at a threshold of *p* < 0.05 and *p* < 0.10 for the quantile regression in order to avoid missing potentially meaningful associations. The statistical analyses for this study were executed using the SPSS-20 and R programming languages.

## 3. Results

A total of 30 metabolically healthy and 32 metabolically unhealthy participants were included in this study. The mean age of the participants was 36.56 ± 7.88 and 38.77 ± 9.44 years, respectively, with 50% and 73.30% of the groups being female. The demographic and clinical characteristics of the metabolically healthy and unhealthy groups are presented in Table 1.

The primary endpoint of this study was to investigate potentially significant differences in the follistatin levels between the different groups. The findings indicated that there was no statistically significant variance in the follistatin levels between the metabolically unhealthy individuals’ median: 41.84 (IQR: 42.41) and their metabolically healthy counterparts’ median: 42.44 (IQR: 43.01) (*p* > 0.05).

Conversely, the metabolically healthy group exhibited a significantly elevated median HDL cholesterol value of 52.50 mg/dL (IQR: 13 mg/dL) compared to the metabolically unhealthy group, which displayed a value of 38.81 ± 6.89 mg/dL (*p* < 0.001). Notably, the triglyceride level in the metabolically unhealthy group, 178 mg/dL (IQR, 143–251.50), was found to be statistically higher than that in the metabolically healthy group, which registered at 96.17 ± 27.12 mg/dL (*p* < 0.001). It was observed that the metabolically unhealthy group exhibited a significantly elevated C-peptide value of 3.23 ng/mL (IQR: 1.37 ng/mL) compared to the metabolically healthy group, which demonstrated a value of 2.71 ± 0.77 ng/mL (*p* = 0.003). Similarly, the AST level in the metabolically unhealthy group (median: 20 U/L (IQR: 12.50)) was significantly higher than that in the metabolically healthy group (18.50 ± 6.20 U/L) (*p* = 0.026).

Multivariate linear regression analysis was performed to investigate the variables that influenced the follistatin levels. Non-significant variables were removed from the model by backward elimination. The final model is presented in Table 2.

The adjusted R-squared value was 38%. The F-statistic was also significant (*p* < 0.001), indicating that the overall model was statistically significant. According to the results, the variable with the most significant impact on the follistatin levels was insulin, with a coefficient of 0.903. This finding implies that an increase in the insulin value by one standard deviation would result in a 0.903 standard deviation increase in the follistatin level. C-peptide had the second most important and negative impact on the follistatin levels, with a coefficient of −0.624. Furthermore, our investigation revealed that the thrombocyte value, fasting blood glucose (FBG) level, LDL cholesterol, and uric acid levels demonstrated a statistically significant and negative effect on the follistatin levels.

To observe the effects of the interaction terms, a regression model was constructed using individual and binary interaction terms. The regression model was adjusted for participant age, and the stepwise regression yielded 15 significant variables, potentially leading to overfitting. To address this, we applied a LASSO regression model for feature selection, eliminating unnecessary or highly correlated variables. The resulting model is summarized in Table 3.

The addition of the interaction terms to the model resulted in a substantial increase in the adjusted R-squared value, from 38% to 56%. The analysis revealed that age and HDL cholesterol had a negative influence on the follistatin levels, with decreases of 0.329 and 0.550 standard deviations, respectively, for every one standard deviation increase. Furthermore, the interactions of HDL cholesterol with age, uric acid and insulin showed statistically significant and complex effects on the follistatin levels. Conversely, the interaction effect of C-peptide and uric acid, as well as the individual effect of C-peptide, had a strong and positive impact on the follistatin levels. Additionally, the interactions between LDL cholesterol and C-peptide, as well as between the FBG and LDL cholesterol levels and various variables, demonstrated intricate associations that contributed to the variation in the follistatin levels. Overall, these findings highlight the intricate interplay between the biological markers and their combined effects on the follistatin levels, providing valuable insights into the potential contributors to the variation in the examined population.

Quantile regression analysis was performed to explore the relationship between follistatin and its potential predictors (thrombocytes, FBG, LDL cholesterol, C-peptide, insulin, and uric acid), providing nuanced insights at diverse quantile levels.

Table 4 presents the outcomes of the quantile regression model, with the quantile levels specified as 0.1, 0.2, ……, 0.8, and 0.9. All the variables, except insulin, demonstrated a negative association with follistatin.

**Figure 1:**
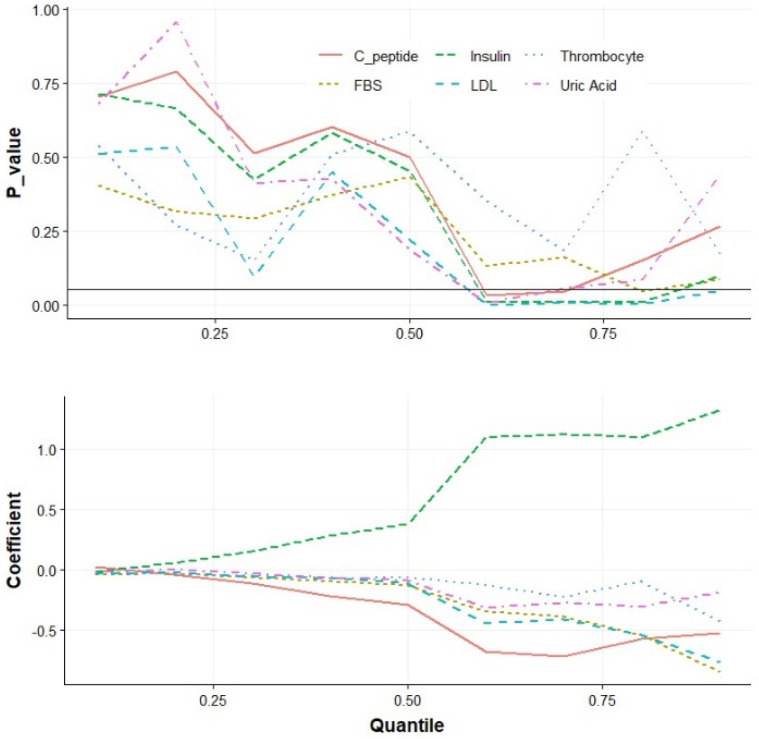
Regression coefficients and *p*-values across various quantiles.

The *p*-values of these variables varied across the quantiles, providing a comprehensive understanding of the statistical significance of their effects. In particular, the thrombocyte values did not exhibit a statistically significant effect across all the quantiles. FBG was negatively and statistically significantly associated with quantiles higher than 0.70 quantile (*p* < 0.1), indicating a significant impact in the upper range of the follistatin values. In other words, a 1% decrease in the FBG value led to an approximately 54% and 85% increase in the follistatin levels in the 0.80th and 0.90th quantiles, respectively. Similarly, the effect of LDL cholesterol was statistically valid and was negative in the quantiles greater than 0.5 (*p* < 0.1). The C-peptide demonstrated a limited effect, primarily observed between the quantile values of 0.6 and 0.7 (*p* < 0.1). The insulin levels were positively and statistically significantly associated with the quantiles higher than 0.5 (*p* < 0.1), suggesting that the insulin level has the most positive impact on the follistatin levels in the range of the 0.6 and 0.9 quantiles. Uric acid exhibited effectiveness in the quantiles between 0.6 and 0.8 (*p* < 0.1). The increase in the regression coefficients of the variables after the 0.5 quantile, as illustrated in Figure 1, adds a dynamic dimension to the analysis, suggesting that the impact of these predictors intensifies beyond the median point in the distribution of the follistatin values.

## 4. Discussion

Follistatin is known for its important role in adipogenesis and metabolic regulation, and it has been hypothesized that it could serve as a biomarker to distinguish metabolic differences in individuals with obesity. Despite growing interest in metabolically healthy obesity (MHO) as a distinct phenotype, our results suggest that the follistatin levels do not differ significantly between metabolically healthy and unhealthy individuals. This challenges the notion that follistatin can be used as a biomarker to distinguish between these two groups in the obese population.

Interestingly, while the follistatin levels showed no discriminatory power between metabolically healthy and unhealthy obesity, other biochemical markers, such as HDL cholesterol, triglycerides, C-peptide, and AST, showed significant differences between these two groups. In particular, individuals with metabolically healthy obesity (MHO) had significantly higher HDL cholesterol levels, whereas those with metabolically unhealthy obesity (MUO) had higher triglyceride, ALT, and C-peptide levels. The HDL cholesterol and triglyceride levels, in particular, are among the criteria for the diagnosis of metabolic syndrome, whereas other values indicate metabolic problems. These findings highlight the multifactorial nature of metabolic health and emphasize the importance of considering various biomarkers for the assessment of obesity-associated metabolic disorders.

One reason for the lack of significant differences in the follistatin levels between metabolically healthy and unhealthy groups could be the inherent complexity and heterogeneity of metabolic disorders associated with obesity [15]. Metabolically healthy obesity (MHO) has attracted considerable attention in recent years and has led to an ongoing debate regarding its clinical significance and long-term prognosis [5]. The assertion that obesity can exist without concomitant metabolic abnormalities challenges conventional concepts and emphasizes the necessity for more comprehensive approaches to describe metabolic health in individuals who are obese.

Inconsistencies in the definition of MHO have made it difficult to compare studies and have further fueled the debate on these individuals. To address this problem, a unified definition of the MHO phenotype has been proposed to standardize the concept [7]. The categorization of individuals as metabolically healthy or unhealthy is based on established criteria such as the presence of components of metabolic syndrome. However, these criteria may oversimplify the intricate interactions between the various pathophysiological mechanisms of metabolism and may not fully capture the extent of the metabolic dysfunction associated with obesity.

Furthermore, the prevalence of MHO varies considerably across populations, suggesting that metabolic health status may be influenced by genetic, environmental, and lifestyle factors that have not yet been fully elucidated or integrated into the existing models [7].

Obesity contributes to the development of chronic inflammation [16]. Adipokines play a significant role in the context of obesity as they act as signaling proteins produced by adipose tissue. These proteins regulate numerous processes, such as inflammation, energy regulation, lipid and glucose metabolism and insulin sensitivity. In obesity, there is an imbalance in the production and release of adipokines, with pro-inflammatory adipokines being activated and anti-inflammatory adipokines being suppressed. This imbalance leads to chronic low-level inflammation and metabolic problems associated with obesity [17,18,19]. The modulation of the production of pro- and anti-inflammatory cytokines by immune cells can regulate inflammatory reactions [16]. A key factor in determining metabolic health in obesity appears to be the balance between pro-inflammatory and anti-inflammatory adipokines.

Studies have shown the following with regard to adipokines. MHO individuals generally have higher levels of adiponectin than MUO. Adiponectin is an anti-inflammatory hormone that improves insulin sensitivity [19]. Leptin levels may be similar or slightly lower in MHO than in MUO, although the body fat levels are comparable. Leptin is considered an inflammatory hormone. Resistin, which is associated with insulin resistance, may be lower in MHO individuals than in MUO individuals [2,15,18].

In one study, the levels of circulating follistatin-like protein1 were significantly elevated in subjects with the MU condition, regardless of whether they were obese or not, but not in subjects with metabolically healthy obesity [20]. In an animal experiment, it was demonstrated that administration of follistatin alone, carried by an adeno-associated virus, resulted in improved muscle function and stimulated white adipose tissue browning [21]. This combined effect appeared to reduce the metabolic inflammation associated with obesity. These results were further supported by changes in the metabolic profile, which indicated lower levels of triglycerides, glucose, free fatty acids and cholesterol in the blood. Taken together, these results led to the investigation of the follistatin levels in metabolically healthy and unhealthy obese patients. The results of this evaluation showed that the follistatin levels did not represent a significant difference between individuals with metabolically healthy obesity and those with metabolically unhealthy obesity.

When performing multivariate linear regression analysis to gain a deeper understanding of the factors affecting the follistatin levels, insulin was found to play the most important role in determining these levels. Additionally, a positive and statistically significant relationship was observed between insulin and the follistatin levels in the higher quantiles. In humans, follistatin is primarily secreted by the liver, and studies have shown that insulin inhibits follistatin expression in hepatocytes [22,23]. Our results are consistent with those of previous studies, suggesting that insulin may have a regulatory effect on follistatin levels. Follistatin is regulated by glucose and can prospectively predict metabolic improvements observed after bariatric surgery [24]. In a study by Sylow et al., the fasting plasma follistatin levels were strongly correlated with fasting hyperinsulinemia and insulin resistance in patients with type 2 diabetes [22].

In the current study, it was observed that the interactions between age, C-peptide levels, LDL cholesterol, uric acid, and insulin could modulate the follistatin levels. This highlights the interaction between these biomarkers and their collective impact on follistatin levels, and it provides valuable insights into the intricate network of metabolic pathways that effectively regulate follistatin. Quantile regression analysis provided further insight into the nuanced relationship between follistatin and its predictors at different quantile levels. Although most variables exhibited a negative association with follistatin, insulin was an exception, with a positive and statistically significant association observed in the higher quantiles.

The observed associations between the follistatin levels and metabolic parameters, such as glucose, insulin, and C-peptide, emphasize the potential role of follistatin in glucose homeostasis and insulin sensitivity. These results are consistent with those of previous studies that have demonstrated positive associations between follistatin levels and markers of insulin resistance and adipose tissue dysfunction. In a study by Perakakis et al., the follistatin levels decreased significantly after oral or intravenous glucose administration [25]. The circulating follistatin levels were lower after bariatric surgery, particularly after Roux-en-Y gastric bypass, and the reduction in the circulating follistatin levels was associated with improved glucose homeostasis and body fat mass after surgery [24].

One of the strengths of this study is the multivariate analysis approach, which indicated that insulin was a significant predictor of follistatin levels. Various other biomarkers, whether in isolation or in combination, have been observed to influence follistatin levels, leading to complex interactions.

In terms of the study limitations, the cross-sectional design limited the ability to infer causal relationships and the relatively small sample size may have affected the statistical power to detect significant differences in the follistatin levels. Furthermore, the fact that the study population originated from a specific geographical area may limit the generalizability of the results. In addition, there could potentially exist unmeasured confounding variables such as dietary patterns, physical activity, stress levels, socioeconomic status, and other lifestyle factors that have the ability to exert an influence on metabolic health and biomarker levels.

Conducting longitudinal studies with larger sample sizes and investigating genetic variations can help to provide a better understanding of the relationship between follistatin levels and metabolic health outcomes. In additional, studying tissue-specific expression patterns and exploring how follistatin affects metabolic parameters in different tissues may provide valuable insights into its role in obesity-related metabolic disorders. Further studies are needed to understand the precise mechanisms underlying the relationship between follistatin, insulin sensitivity, and glucose regulation.

## 5. Conclusions

Although follistatin may not serve as a discriminatory biomarker to distinguish between metabolically healthy and unhealthy obesity, our results emphasize the complexity of metabolic health in individuals who are obese. The limitations of the current classification systems and the necessity for more sensitive methods to assess metabolic health emphasize the need for further research in this area. Ultimately, a deeper understanding of the underlying mechanisms that contribute to metabolic dysfunction in obesity could lead to more personalized and effective strategies for managing obesity and preventing metabolic diseases and the associated complications.

## Figures and Tables

**Table 1:** Demographic and clinical profiles of study participants.

	Metabolically Unhealthy (*n* = 32)	Metabolically Healthy (*n* = 30)	Total (*n* = 62)	*p*-Value
Age (Years)	36.56 ± 7.88 ^a^	38.77 ± 9.44 ^a^	37.63 ± 8.67 ^a^	0.324
Gender (n, %)				
Female	16 (50)	22 (73.30)	38 (0.61)	0.075
Male	16 (50)	8 (26.70)	24 (0.39)	
Smoking Status (n, %)				
Yes	10 (31.25)	8 (26.67)	18 (29.03)	0.799
No	22 (68.75)	22 (73.33)	44 (70.97)	
Diabetes (n, %)				
Yes	3 (9.38)	0 (0)	3 (4.84)	0.798
No	29 (90.62)	30 (100)	59 (95.16)	
Follistatin (NPX)	41.84 (42.41) ^b^	42.44 (43.01) ^b^	41.96 (44.52) ^b^	0.642
Thrombocyte (µU)	291,612.90 ± 57,656.85 ^a^	289,600 ± 53,848.19 ^a^	290,622.95 ± 55,359.48 ^a^	0.888
BMI (Kg/m^2^)	35.31 ± 4.49 ^a^	37.47 ± 7.12 ^a^	34.90 (6.81) ^b^	0.338
HbA1c (%)	5.70 (0.60) ^b^	5.6 (0.40) ^b^	5.6 (0.50) ^b^	0.102
FBG (mg/dL)	91 (13.50) ^b^	90.80 ± 7.61 ^a^	90 (12) ^b^	0.735
LDL-C (mg/dL)	108.78 ± 28.09 ^a^	113.70 ± 31.29 ^a^	111.16 ± 29.54 ^a^	0.518
HDL-C (mg/dL)	38.81 ± 6.89 ^a^	52.50 (13) ^b^	44.5 (13.50) ^b^	0.000 *
Triglyceride (mg/dL)	178 (108.50) ^b^	96.17 ± 27.12 ^a^	121.50 (91.50) ^b^	0.000 *
Total Cholesterol (mg/dL)	173.50 (51.50) ^b^	188.53 ± 33.50 ^a^	178 (47) ^b^	0.667
C-peptide (ng/mL)	3.23 (1.37) ^b^	2.71 ± 0.77 ^a^	3.08 (0.91) ^b^	0.003 *
Insulin (µU/mL)	19.57 (15.15) ^b^	15.98 ± 7.37 ^a^	18.25 (10) ^b^	0.119
Urea (mg/dL)	25.75 ± 7.26 ^a^	25.86 ± 6.16 ^a^	25.80 ± 6.70 ^a^	0.949
Creatinine (mg/dL)	0.79 ± 0.17 ^a^	0.74 (0.22) ^b^	0.74 (0.22) ^b^	0.077
Uric Acid (mg/dL)	5.24 ± 1.21 ^a^	4.74 ± 1.03 ^a^	4.99 ± 1.14 ^a^	0.087
TSH (mIU/L)	2.13 ± 1.06 ^a^	2.17 ± 1.15 ^a^	2.15 ± 1.10 ^a^	0.894
FT4 (ng/dL)	1.17 (0.22) ^b^	1.16 ± 0.14 ^a^	1.17 (0.18) ^b^	0.978
AST (U/L)	20 (12.50) ^b^	18.50 ± 6.20 ^a^	19 (9) ^b^	0.026 *
ALT (U/L)	23.50 (20.50) ^b^	18.50 (14.31) ^b^	20.50 (19) ^b^	0.181

Continuous variables (^a^) are presented as the mean ± standard deviation for normally distributed or median (IQR) for non-normally distributed (^b^). Categorical variables were presented as numbers, percentages. * Statistical significance at *p* < 0.05. BMI = body mass index; HbA1c = glycated hemoglobin; FBG = fasting blood glucose; LDL = low-density lipoprotein; HDL = high-density lipoprotein; TSH = thyroid-stimulating hormone; FT4 = free thyroxin; AST = aspartate aminotransferase; ALT = alanine transaminase.

**Table 2:** Multiple regression analysis results using the backward elimination method.

Dependent Variable	Independent Variables	Standardized Coefficients	Standard Error	*t*-Value	*p*-Value
Follistatin Level	Thrombocyte	−0.216	0.121	−2.059	0.044
	FBG	−0.385	0.131	−2.915	0.005
	LDL-C	−0.404	0.106	−3.812	0.000
	C-peptide	−0.624	0.226	−2.759	0.008
	Insulin	0.903	0.241	3.735	0.000
	Uric Acid	−0.351	0.114	−3.111	0.003

Abbreviations: FBG: fasting blood glucose; LDL-C: LDL cholesterol.

**Table 3:** Multiple regression model with interaction terms.

Dependent Variable	Independent Variables	Standardized Coefficients	Standard Error	*t*-Value	*p*-Value
Follistatin Level	Age	−0.171	0.112	−1.521	0.034
	HDL-C	−0.632	0.161	−3.914	0.001
	HDL-C: Insulin	−0.733	0.2014	−3.639	0.000
	C-peptide	0.453	0.143	−3.174	0.002
	C-peptide: Uric Acid	0.316	0.150	2.101	0.040
	LDL-C: Uric Acid	0.278	0.125	2.214	0.031

Abbreviations: HDL-C: HDL cholesterol; FBG: fasting blood glucose; LDL-C: LDL cholesterol.

**Table 4:** Quantile regression estimation results.

	Thrombocyte		FBG		LDL-C		C-Peptide		Insulin		Uric Acid	
Quantile	Coefficient	*p*-Value	Coefficient	*p*-Value	Coefficient	*p*-Value	Coefficient	*p*-Value	Coefficient	*p*-Value	Coefficient	*p*-Value
0.100	−0.021	0.538	−0.045	0.405	−0.036	0.509	0.024	0.704	−0.021	0.711	−0.016	0.680
0.200	−0.038	0.270	−0.037	0.317	−0.030	0.536	−0.039	0.789	0.054	0.664	−0.002	0.957
0.300	−0.054	0.151	−0.063	0.291	−0.061	0.095	−0.114	0.512	0.150	0.422	−0.034	0.410
0.400	−0.072	0.509	−0.101	0.370	−0.072	0.449	−0.219	0.601	0.278	0.582	−0.069	0.426
0.500	−0.063	0.584	−0.128	0.433	−0.119	0.218	−0.289	0.501	0.373	0.452	−0.092	0.188
0.600	−0.133	0.350	−0.349	0.130	−0.446	0.001	−0.682	0.033	1.092	0.010	−0.314	0.006
0.700	−0.229	0.182	−0.388	0.161	−0.411	0.006	−0.716	0.044	1.122	0.010	−0.278	0.053
0.800	−0.098	0.588	−0.541	0.045	−0.539	0.001	−0.574	0.149	1.095	0.007	−0.308	0.085
0.900	−0.428	0.169	−0.848	0.083	−0.766	0.050	−0.524	0.265	1.322	0.096	−0.191	0.439

Abbreviations: FBG: fasting blood glucose; LDL-C: LDL cholesterol.

## Data Availability

The raw data supporting the conclusions of this article will be made available by the authors on request.
